# ASO Author Reflections: Rethinking Lymphedema Prophylaxis After Groin Dissection for Melanoma

**DOI:** 10.1245/s10434-026-19697-9

**Published:** 2026-04-28

**Authors:** Melody Brown, Teresa S. Lee, Isabel Li, Christopher Allan

**Affiliations:** 1 Mater Hospital, Brisbane, Australia; 2 Royal North Shore Hospital, St Leonards, Australia; 3 University of Sydney, Camperdown, Australia; 4 NHMRC Clinical Trials Centre, Sydney, Australia

## Past

Lower limb lymphedema is a common and troublesome complication following groin dissection for metastatic melanoma, affecting approximately one third of patients.^1^ Traditionally, management has been reactive, with treatment initiated after clinical onset. Evidence from breast cancer has supported early surveillance and prophylactic compression to reduce progression to chronic lymphedema,^2,3^ but data in melanoma patients after groin dissection are limited.

## Present

In this context, our randomized trial of 38 participants explored whether early prophylactic compression garments combined with simple lymphatic drainage (CG-SLD) could alter the trajectory of leg lymphedema development compared with standard care (SC) in melanoma patients. During the active intervention period, lymphedema incidence was substantially lower with CG-SLD (3 months: 11.1 vs. 45%; 6 months: 16.7 vs. 50%), accompanied by reduced early severity.^4^ However, this benefit was not sustained once the intervention ceased. By 12 months, differences between groups had narrowed, with no new cases thereafter, and at 24 months, overall lymphedema incidence remained high (47.4%) with no statistically significant difference between groups (SC: 55% vs. CG-SLD: 38.9%, *p* = 0.4). These findings suggest that early compression may delay, rather than prevent, lymphedema. The study’s limited sample size and attrition further constrain definitive conclusions but do not diminish the clinical effect observed.

## Future

These findings prompt a shift in thinking from whether to intervene early to how long early intervention should be sustained. Extending the duration of prophylactic strategies may be necessary to support lymphatic recovery and maintain early gains. A risk-stratified approach may help target patients most likely to benefit from prolonged therapy, particularly those exposed to additional risk factors, such as nodal radiation. Future studies should also incorporate patient-reported outcomes to better capture the trade-offs between efficacy and burden. Ultimately, determining whether longer-duration intervention can translate into sustained reductions in lymphedema burden remains a key priority.
